# Innovative Progress of LSPR‐Based Dark‐Field Scattering Spectral Imaging in the Biomedical Assay at the Single‐Particle Level

**DOI:** 10.1002/open.202400017

**Published:** 2024-12-27

**Authors:** Yang Shi, Lixiang Wang, Lingling Li, Chen Feng, Yue Cao

**Affiliations:** ^1^ Department of Forensic Medicine Nanjing Medical University Nanjing 211166 PR China; ^2^ School of Pharmacy Nanjing Medical University Nanjing 211166 PR China

**Keywords:** Dark-field microscopy, Scattering imaging, Single particle, Localized surface plasmon resonance, Bioassay

## Abstract

The growing demand for detection and sensing in the biomedical field is placing higher demands on technology. In clinical testing, it is expected to be able to realize both rapid large‐field imaging and analysis of single particles (or single molecules or single cells), and it is expected to be able to grasp both the unique individuality of single particles in time and space during the complex reaction process, as well as the regular correlation between single particles in the same population distribution. Supported and promoted by the theory of localized surface plasmon resonance (LSPR), dark‐field microscopy, as a single‐particle optical imaging technique with a very high signal‐to‐noise ratio, provides a powerful new means to address the above clinical detection needs. This review will focus on the innovative applications of dark‐field microscopy in biomedical‐related assays in the past five years, introducing the basic principles and listing the impressing works. We also summarize how dark‐field microscopy has been combined with other techniques, including surface‐enhanced Raman scattering, fluorescence, colorimetry, electrochemistry, etc., to witness the joint progress and promotion of detection methods in the future. It also provides an outlook on the current challenges and future trends in this field.

## Graphical abstract

This review explores recent advancements in dark‐field microscopy for biomedical detection, focusing on innovative applications in the past five years. It examines basic principles, notable works, and integration with other techniques like Raman scattering and fluorescence. The study highlights dark‐field microscopy's potential in addressing clinical needs for rapid imaging and single‐particle analysis in complex biological systems, discussing current challenges and future trends.

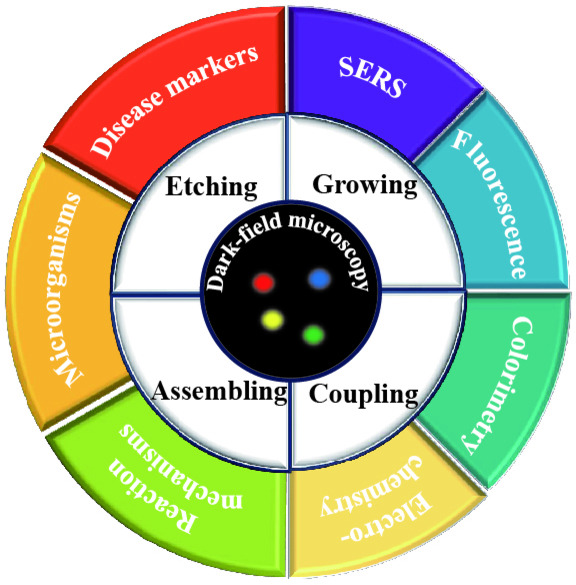

## 1 Introduction

Since the 17th century, the science of microscopy has developed rapidly and steadily. Microscopes are now indispensable for many scientific studies worldwide, and their types can be broadly categorized into optical and electron microscopes. Each type of microscope has its most suitable application: common optical microscopes are used for general imaging and detection of various microorganisms, while electron microscopes can be used to characterize and identify the morphology and chemical composition of the nanomaterials being investigated.^[1]^ Despite these microscopes offer definite advantages, they also have some limitations. For example, optical microscopy is unable to detect particles smaller than 200 nm in size due to its diffraction‐limited resolution,^[2]^ while electron microscopy is costly and requires specialized personnel skilled in its operation. Therefore, there is a need for an alternative microscopy technique that is simple, fast and non‐destructive for in situ imaging of single particles at the nanoscale.

Dark‐field microscopy, as a specific subtype of optical microscopy, is essentially the clear optical imaging of objects against a dark‐field background.^[3]^ Due to its unique configuration, dark field microscopy enables fast, non‐destructive, real‐time and inexpensive imaging of nanoparticles. Combined with a companion spectrometer, it is possible to obtain specific optical information about the color, number, spectral peak position and intensity of individual particles in the field of view.^[4]^ It is possible to break through the limitation of the spatial resolution of about 200 nm of conventional optical microscopes.^[5]^

Combined with various types of cleverly designed nanoparticles, dark‐field microscopy has been widely utilized and advanced in sensing analysis, imaging, monitoring chemical reaction processes, and revealing reaction mechanisms.

In recent years, nanomaterial science has developed rapidly. Nanomaterial science can be interdisciplinary with physics, biology, medicine and other disciplines. It has received extensive attention and research in various fields, and its preparation and application have been greatly improved and broken through. Among them, there are many free electrons on the surface of noble metal (gold, silver) nanomaterials.^[6]^ Under the irradiation of white light, free electrons are excited and strongly resonate and scatter with the incident light, and are able to resonate with electromagnetic waves of specific frequencies, which is called the localized surface plasmon resonance effect^[7]^ (Localized surface plasmon resonance, LSPR).Materials with this effect are called Plasmonic nanoparticles (PNPs). According to the Mie scattering absorption theory equation,^[8]^ their optical properties can be modulated by the size, shape, composition, surrounding dielectric environment, charge density, etc. of the nanoparticles.^[9]^

As a result, researchers have begun to explore noble metal nanomaterials, and their shapes have evolved from simple spherical nanoparticles to rods, disks, stars, cones, chips, cubes, and other shapes, and from a single structure to multi‐dimensional and composite nanostructures.^[10]^ Dark‐field imaging of their scattered light also has specific colors and corresponding spectra, and the noble metal nanoparticle‐based probes are able to reflect in real time the changes in the probe itself and the surrounding environment during the target reaction process,^[11]^ with higher sensitivity, faster response time and broader potential for detection and sensing applications.

## 2 Principles, Instrumentation and Methodology

### 2.1 Principles of LSPR

**Figure 1:**
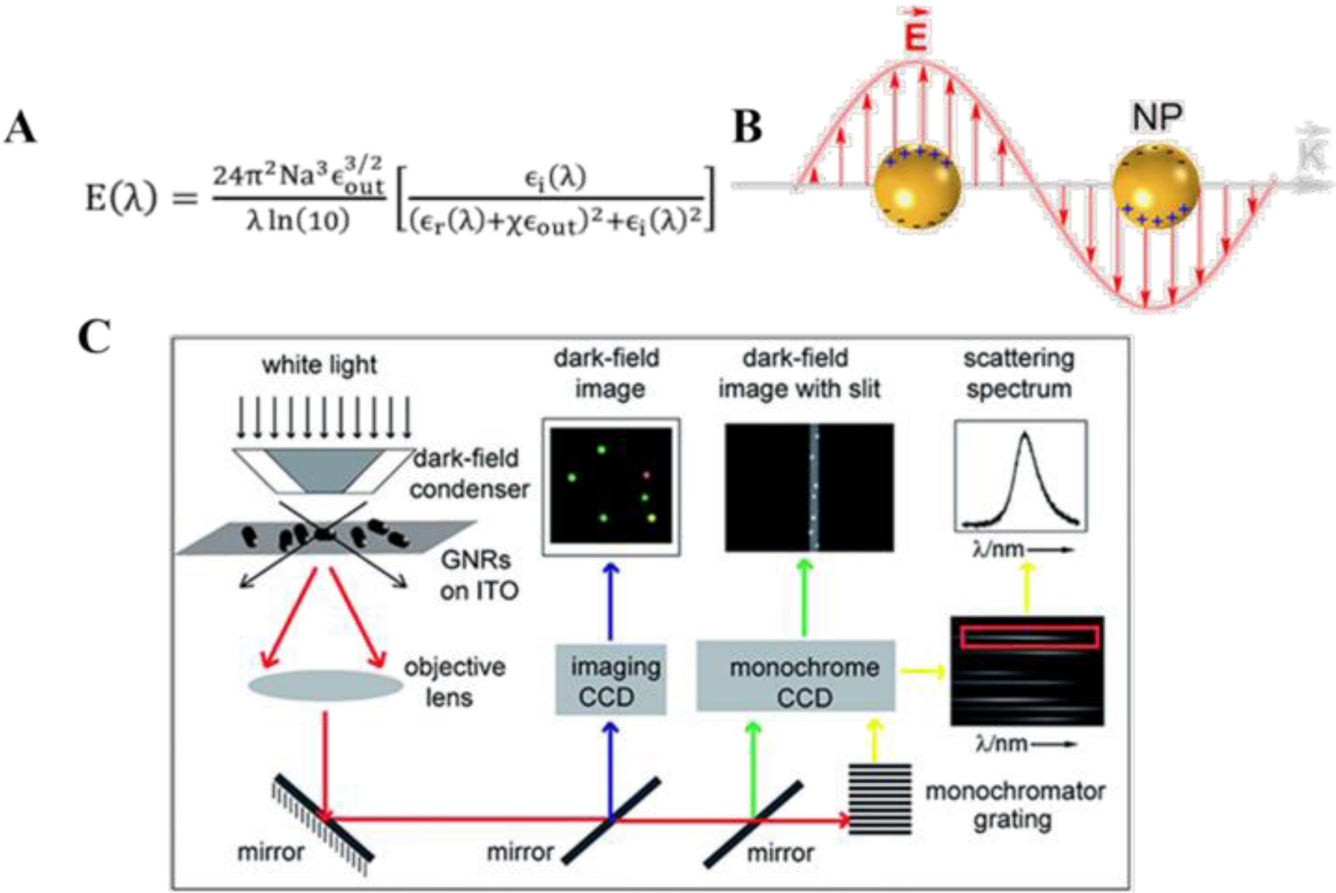
A): Mie theory formula for LSPR absorption or scattering spectra^[13]^ B): Principle of localized surface plasmon resonance. Reprinted with permission from Jauffred, L., Samadi, A., Klingberg, H., Bendix, P. M. & Oddershede, L. B. Chem. Rev. 119, 8087–8130 (2019).^[14]^ Copyright 2019 American Chemical Society. C):Schematic structure of the dark‐field scattering imaging technique. Reprinted with permission from Jing, C., Shi, L., Liu, X. & Long, Y.‐T. Analyst 139, 6435–6439 (2014).^[19]^ Copyright 2014 ROYAL SOCIETY OF CHEMISTRY.

Localized surface plasmon resonances are nanoscale phenomena that have received much attention in recent years. Resonances are associated with nanostructures, producing sharp absorption and scattering peaks and strong electromagnetic field enhancements.[^12^, ^13^] During the last two decades, there have been significant advances in the preparation of nobel metal nanomaterials, which have contributed to the progress of the field of LSPR. In 1908, Mie provided a theoretical explanation for the scattering of spherical nanoparticles.^[14]^ Free electrons in metals are able to oscillate in concert with an incident oscillating electromagnetic field.^[15]^ When a light source of a certain wavelength is incident on a metal nanoparticle, if the incident photon frequency matches the overall vibrational frequency of the metal nanoparticle, the metal nanoparticle will have a strong absorption of photon energy at this time, which exhibits a strong resonance absorption peak on the spectrum (Figure 1B), thus generating the LSPR phenomenon.^[16]^ The total cross section consisting of SPR absorption and scattering is the sum of all electromagnetic oscillations according to the Mie theory, which is an approximate solution to the set of Maxwell's equations (Figure 1A) and quantitatively describes the effect of LSPR.

### 2.2 Dark‐Field Scattering Imaging Technique

Usually dark‐field microscopes are divided into two types: orthogonal and inverted, taking the typical inverted one as an example (Figure 1C): a parallel beam of white light passes through a light barrier that cuts off the center portion of the incident light. No light enters the objective lens at this point. The incident light at the edge of the ring is converted into a hollow cone of light by the optics in the dark‐field concentrator with a higher numerical aperture, causing the white light to converge on the surface of the nanoparticle.^[17]^ After the nanoparticles are excited, an objective lens with a numerical aperture smaller than that of a condenser lens is used underneath to collect only the scattered light of the sample, and the optical signal is transmitted to the dark‐field imaging CCD through the microscope system, the entire background of the view appears black, and only the scattered light spots of the nanoparticles appear bright. Also, the scattering spectra of the sample can be obtained through the slit to reach the spectrometer.^[18]^

Dark field microscopy enables small‐sized nanoparticles to be clearly imaged.^[19]^ The resolution is significantly higher than that of ordinary microscopes, overcoming the problem of insufficient transmission or reflection contrast in conventional optical microscopy, and the nanoparticles are able to stand out against dark backgrounds. Therefore, dark‐field microscopy has the outstanding advantages of high signal‐to‐noise ratio, high sensitivity, and high spatial and temporal resolution.^[20]^

## 3 Innovations in Probe Morphology

**Figure 2:**
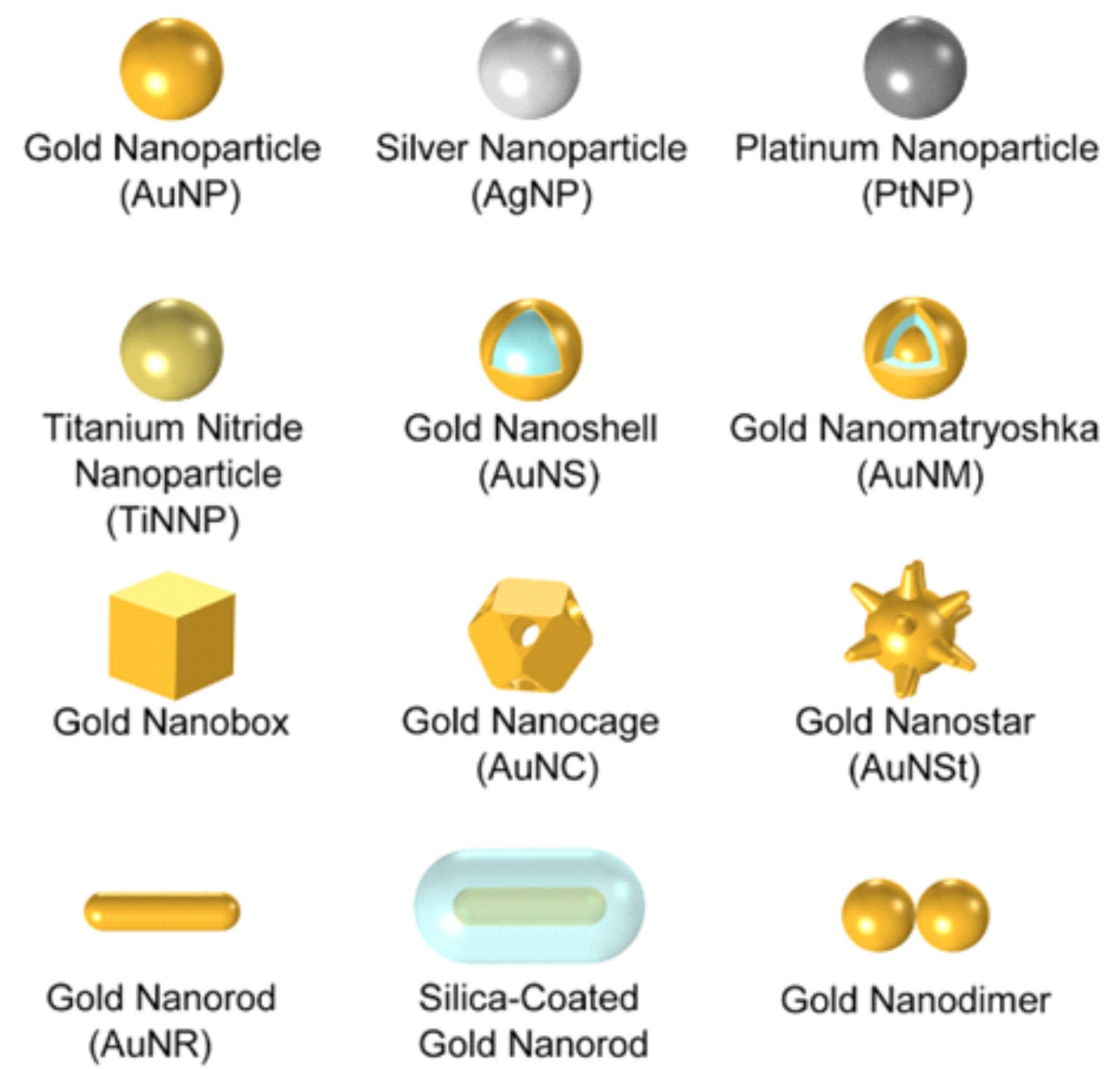
Common morphology and structure of nano‐plasmas. Reprinted with permission from Jauffred, L., Samadi, A., Klingberg, H., Bendix, P. M. & Oddershede, L. B. Chem. Rev. 119, 8087–8130 (2019).^[14]^ Copyright 2019 American Chemical Society.

Mie theory provides a theoretical explanation for the LSPR of spherical nanoparticles, and although there is no precisely applicable theoretical model for the LSPR sensitivity of non‐spherical noble metal nanoparticles, experiments and simulations have shown that the particle morphology plays a great role in determining sensitivity.[^15^, ^21^] Dark‐field microscopy can not only realize single‐particle imaging,^[22]^ but the obtained scattering spectra are also widely used in the study of scattering properties of noble metal nanoparticles, surface nanostructure research, etc., which can obtain information on the scale, state, kinetic process and interaction with the surrounding environment of nanoparticles.^[23]^ The easily tunable morphology of precious metal nanoprobes is closely related to their scattering optical properties. Therefore, in order to realize the broader application prospect of dark‐field microscopy, in the past two decades, researchers have developed countless new nanomaterials with different compositions and morphologies, and excellent refractive indices and sensitivities, such as nanoparticles of gold, silver^[24]^ and platinum, forming nanospheres, nanorods, nanocubes, nanostars, nanobipyramids and other structures,^[25]^ some of them have core‐shell sandwich structure, some have hollow mesoporous structure, and some are polymer(Figure 2).^[26]^ Different morphologies of nanoparticles exhibit significant variations in these properties. Spherical nanoparticles typically display a single surface plasmon resonance (SPR) peak that redshifts with increasing particle size.^[27]^ In contrast, rod‐shaped nanoparticles reveal multiple SPR peaks, reflecting their anisotropic shape, with distinct optical characteristics in the longitudinal and transverse directions.^[28]^ Sheet‐like nanoparticles demonstrate pronounced optical anisotropy due to their geometric structure, which may lead to multiple scattering effects, causing their spectral features to vary with different incident angles.^[29]^ The scattering spectra of polyhedral particles are more complex, often exhibiting overlapping SPR peaks due to the presence of multiple faces and angles, with significant surface effects.[^30^, ^31^] Irregularly shaped nanoparticles present broad spectral widths and uncertainties, resulting from their random configuration, which leads to mixed optical characteristics. Understanding the scattering spectral properties of these different morphologies is essential for sensor design, optical imaging, and catalytic performance optimization. This knowledge facilitates advancements in applications across fields such as biomedicine, optoelectronics, and environmental monitoring. By analyzing the relationship between nanoparticle morphology and optical properties, we can provide a theoretical foundation for the design of new materials, thereby unlocking their broader application potential.

## 4 Sensing Mechanism

Currently, the detection strategy in analytical sensing is mainly to cause changes in the optical properties of noble metal nanoparticles either directly or indirectly, which mainly includes: etching, growing,assembling, coupling and other strategies.^[32]^

### 4.1 Etching

In previous reports, researchers have realized the detection of a variety of biomarkers by etching. The commonly used etching agents are also diverse, such as common H_2_O_2_, enzymes, TMB^2+^, and iodine monomers. And etching often causes changes in the scattering intensity of the probe, shifts of the scattering spectrum with color change in dark‐field imaging.

Huang's group used dark‐field imaging to monitor in real time the etching process of three shapes of gold nanoprobes under the action of KI/I_2_, namely gold nanospheres, gold nanorods, and gold nanotriangular sheets.^[33]^ During the oxidative etching process, the gold nanoparticles with different morphologies undergo significant scattered light color changes, blue shifts of the scattering spectrum, and decreases in the scattered light intensity. Both time‐domain finite‐difference (FDTD) simulations and monitoring of morphology changes demonstrate that oxidative etching of gold nanoparticles is a thermodynamically dependent chipping reaction. Wang et al. used a gold nanotriangular sheet as a probe with HRP bound to it. In the presence of H_2_O_2_, HRP catalyzed the change of Br‐ to Br^⋅^, which etched away the tip of the gold nanotriangular sheet and changed it into a disc shape, causing blue shifts of the spectrum and changes of the color,^[34]^ which enabled the visualization and rapid sensing detection of hydrogen peroxide.

### 4.2 Growing

Xu et al. reduced Pt, Pd, and Rh in the outer layer of gold nanocores by the catalytic activity of methanol oxidation reaction. The surface deposition process and the formation of the core‐shell structure could be traced in real time and accurately by the dark‐field scattering colors, intensities, and wavelengths.^[35]^ The metals deposited on the surface of gold spheres have good catalytic activity, and when combined with the plasmon resonance effect, they greatly enhance the electrocatalytic activity of MOR, which has good prospects for general application. The group also has similar work to synthesize metal@semiconductor core‐shell nanocrystals by selectively depositing CdS, CdSe and ZnS on the surface of Au cores.^[36]^ New directions have been opened in the utilization of visible light and improvement of photocatalytic efficiency. Lin et al. used Au@AgI core‐shell nanoparticles as probes for the rapid monitoring of sulfide in biological environments.^[37]^ When Au@ AgI PNPs were exposed to sulfide, AgI was converted to Ag_2_S, leading to a change in LSPR, which was sensitively observed under dark‐field microscopy.

### 4.3 Assembling

Yan et al. constructed two nanoprobes by selectively modifying hairpin DNA on the surface of gold nanorods. When microRNA‐ 21 and Let‐7a were present, CHA cycle assembly was triggered to form end‐to‐end (ETE) and side‐by‐side (SBS) dimers, respectively. The scattering colors and intensities were significantly changed compared to a single AuNR. Then, a principal component analysis data processing method was utilized to distinguish the three states of AuNRs, which enabled the sensitive detection of two microRNAs at the same time. In addition, this work enabled intracellular high‐resolution imaging, which also provided valuable information for targeted assembly.^[38]^ Disassembly is also an effective means of sensing. Wang et al. caused the target microRNA to trigger the disassembly of the probe's core‐satellite structure through a strand displacement reaction,^[39]^ resulting in a significant change of the dark field image from red to the green color of the gold core.

### 4.4 Coupling

Liu et al. used 50 nm gold nanoparticles as nuclei on which double‐stranded DNA was modified. in the presence of dsDNA activity, PARP‐1 catalyzed the synthesis of the polymer PAR on the surface of the gold nuclei using coenzyme I as a substrate, which was then electrostatically adsorbed onto the 8 nm‐sized AuNPs.^[40]^ As a result, the localized surface plasmon resonance scattering spectra of Au50 underwent a significant redshift accompanied by an obvious color change.

## 5 Practical Applications of Dark Field Scattering Imaging

The classical basic strategies mentioned above have been widely used in many directions after researchers ′ deformation and innovation, such as quantitative detection of disease markers, classification or quantitative detection of microorganisms, and revealing chemical reaction mechanisms.

### 5.1 Applications Related to Biological Diseases

Biomarkers can be categorized into biochemical markers, molecular markers, cellular markers, imaging markers, immune markers and microbial markers. All kinds of markers have their wide application in clinic, among which biochemical markers are the most important ones that can reflect the dynamic level of human health indicators in real time. Biochemical markers can be categorized into protein markers, nucleic acid markers, lipid markers and carbohydrate markers according to their composition.

#### 5.1.1 Protein Markers

**Figure 3:**
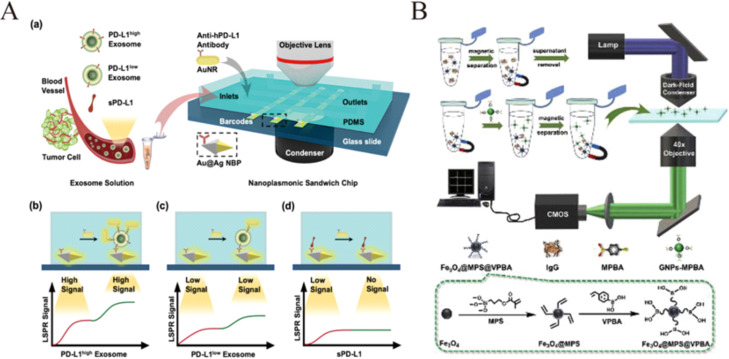
A): Sensing principle of the nanoplasmonic sandwich immunoassay for exosome quantification and subclass identification. Reprinted with permission from Wang, C. et al. ACS Sens. 6, 3308–3319 (2021).^[34]^ Copyright 2021 American Chemical Society. B): Schematic illustration of the GNPs enumeration method for IgG detection and the light path for DFM imaging. Reprinted with permission from Han, Y., Ye, Z., Chen, L. & Xiao, L. Analytica Chimica Acta 1109, 53–60 (2020).^[41]^ Copyright 2020 Elsevier.

Chen's group designed a novel and subtle sensing detection platform to detect exosomes and the expression level of PD−L1 on them by dark‐field scattering intensity using sandwich immunoassays^[41]^ (Figure 3A). On a microfluidic platform, silver‐coated gold bipyramids modified with PD−L1 antibody were used to capture exosomes, and the scattering intensity increased upon binding. Gold rods also modified with PD−L1 antibody were then added. Exosomes with high expression of PD−L1 will receive more scattering intensity increase from the gold rods coupled with the gold biconicals, thus realizing the typing of exosomes.

Xu's group reports a strategy for the detection of the alkaline phosphatase ALP by using an equipartitioned exciton probe with FeOOH encapsulated outside of gold nanoparticles. ALP, as a hydrolase, is capable of hydrolyzing L‐ascorbic acid‐2‐phosphate to ascorbic acid. Ascorbic acid subsequently acts as a strong reducing agent to etch away the FeOOH shell layer, inducing a blue shift in the localized surface plasmon resonance spectra and a pronounced color change under the DFM.^[42]^ RGB numerical analysis of dark‐field images using ΔR/G as the signal output enables fast and sensitive detection of ALP in real time, with detection limits as low as 0.06 U/L. In a similar etching strategy, li et al. designed core‐shell structured Au@Ag nanocubes to detect ALP.^[43]^ In the presence of ALP, AA2P was hydrolyzed to AA by ALP, reducing KIO_3_ to I_2_, which in turn etched the outer silver shell, resulting in a significant color change in dark‐field imaging, with a limit of detection of 0.183 μU/mL. Wang et al. designed GNP@MnO_2_, which also achieved detection of ALP, with a linear range of 0.06 to 2.48 mU/mL.^[44]^

Qi et al. used gold nanoparticles as probes to form GNPs@Cu structures by gold‐catalyzed reduction of NADH. In the presence of PPi, because PPi is preferentially coordinated to Cu^2+^, Cu^2+^ cannot be reduced to Cu^0^, making the gold nanoparticles appear originally green. PPi can be easily quantified by counting the concentration‐dependent fraction of yellow particles representing GNPs@Cu in the DFM image with a detection limit of 1.49 nM.^[45]^ Li et al. also took advantage of the easy formation of a stable structure between PPi and Cu^2+^, and then added sodium ascorbate (NaAsc), and designed a dark‐field imaging sensor based on the click reaction for detecting pyrophosphatase.^[46]^ When pyrophosphatase is present, the stable structure of PPi and Cu^2+^ is disrupted, and Cu^2+^ is released and reduced to Cu^+^ by sodium ascorbate, which catalyzes the azide and alkyne groups on the nucleus and satellite gold nanoparticles, causing the two to couple, resulting in a change of scattering signals in the dark field, and enabling the sensing of PPase in serum and the screening of inhibitors.

Xu's group developed an Au@MnO_2_‐DNA plasmonic material with core‐shell structure for dark‐field and fluorescence dual‐mode imaging of intracellular glutathione.^[47]^ High levels of intracellular glutathione can oxidize the MnO_2_ shell, and both the scattering signal and fluorescence signal changes generated by the probe are correlated with the expression level of GSH. Moreover, Mn^2+^ produced by the reduction of MnO_2_ by glutathione can be used as a chemokinetic reagent to participate in the Fenton‐like reaction, enhancing chemokinetic treatment of cancer cells, and also alleviating the side effects of intracellular GSH in cancer treatment. This material has both diagnostic imaging as well as chemokinetic therapeutic roles.

Han et al. designed borate polymer‐functionalized magnetic nanoparticles(Fe_3_O_4_@MPS@VPBANPs) and 4‐mercaptophenylboronic acid‐modified gold nanoparticles (GNPs‐MPBA) based on boronate affinity interactions, which were connected to each other by the target detector IgG to form a sandwich complex. (Figure 3B). After magnetic separation, the amount of GNPs‐MPBA in solution decreased with increasing IgG. The concentration‐dependent GNPs‐MPBAs were subsequently counted and counted in the dark field to realize the quantification of glycoprotein IgG.^[48]^

#### 5.1.2 Nucleic Acids

**Figure 4:**
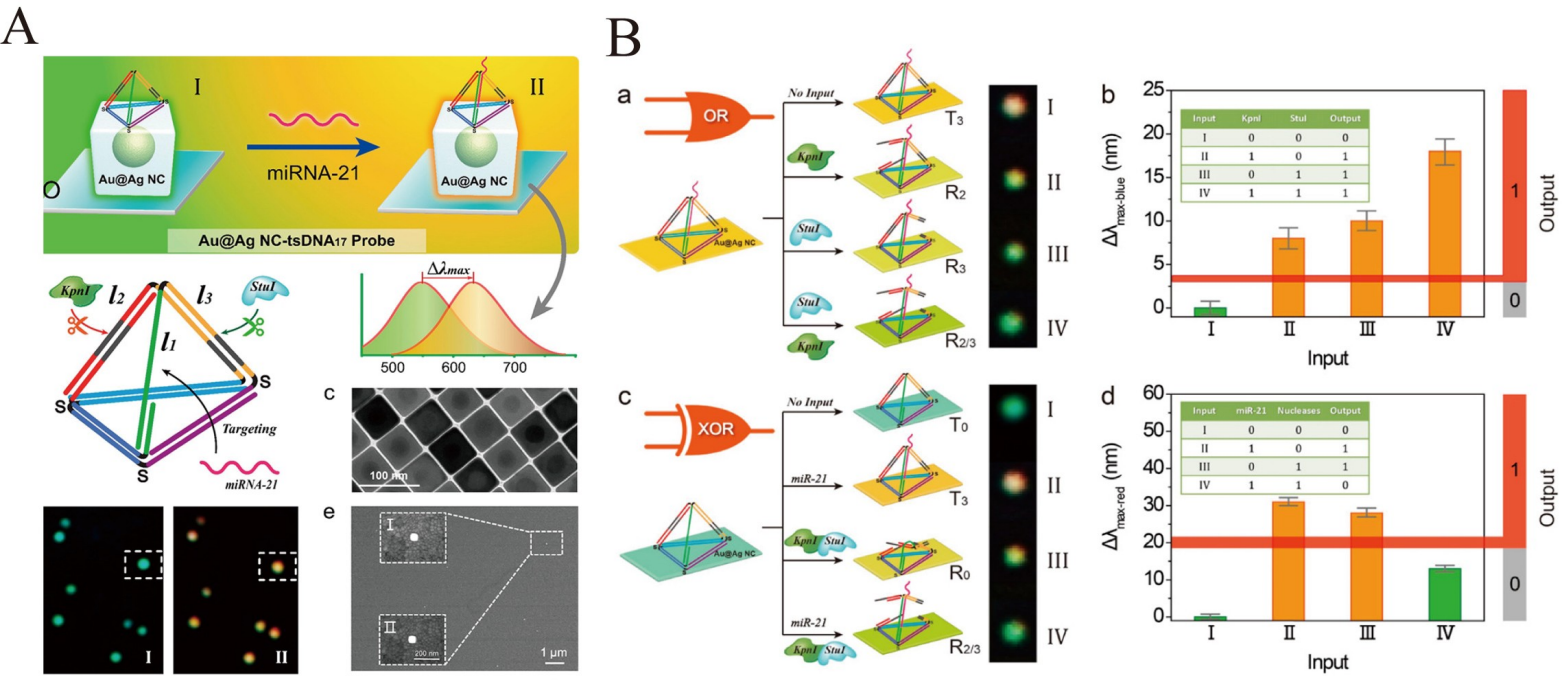
A): Schematic illustration of the detection of miRNA‐21 based on Au@Ag core‐shell nanocube modified by tetrahedral‐structured DNA. Reprinted with permission from Zhang, Y. et al. J. Am. Chem. Soc. 140, 3988–3993 (2018).^[42]^ Copyright 2018 American Chemical Society. B): Schematic representation of OR and XOR gate based on the RI change, and their relative signal output.

Zhang designed a smart sensor based on a single Au@Ag core‐shell nanocube modified by tetrahedral‐structured DNA for the detection of microRNAs at the single‐molecule level^[49]^ (Figure 4A and B). This system not only detects miRNA‐21 in real time, but also dynamically detects miR‐21 in the range of 1 aM to 1 nM. And it can also analyze miR‐21, KpnI and stui‐response based on DNA logic gates of biological memory. A similar probe for Au@Ag NCs was designed by Guo et al. by introducing a large amount of Gox on a glass substrate, and the Gox generates H_2_O_2_ to cause the Au@Ag NCs to etch, resulting in a change of the scattering signal.^[50]^ Based on this change in scattering signal, miRNA‐21 can be detected with high sensitivity with a detection limit of 1.0 fM. Li et al. constructed a rapid and simple method for the detection of Mycobacterium tuberculosis‐specific DNA.^[51]^ Utilizing RCA amplification, a single target molecule can generate hundreds of universal oligonucleotides to form a sandwich structure with capture strand‐modified magnetic beads and AuNPs. After magnetic separation, the AuNPs are released and detected by dark‐field imaging, which is able to distinguish ~10 fM Mycobacterium tuberculosis‐specific DNA from the blank in an assay volume of only 6 μL.

#### 5.1.3 Others

Xiao's group designed a simple gold nanoprobe modified with mercaptophenylboronic acid for the detection of galactose in blood and urine.^[52]^ When galactose is present, the boronic acid monomer MPBA can covalently bond with two cis‐o‐diol groups in the galactose molecule to form stable boronic acid ester bonds. The distance between these MPBA‐GNPs decreases under the galactose linkage, leading to the occurrence of equipartitional exciton coupling. The scattering color of the assembled MPBA‐GNPs changes from green to yellow in the dark field image. Detection of galactose concentration by counting yellow particles in dark‐field images can be used for early detection of metabolic diseases.

**Figure 5:**
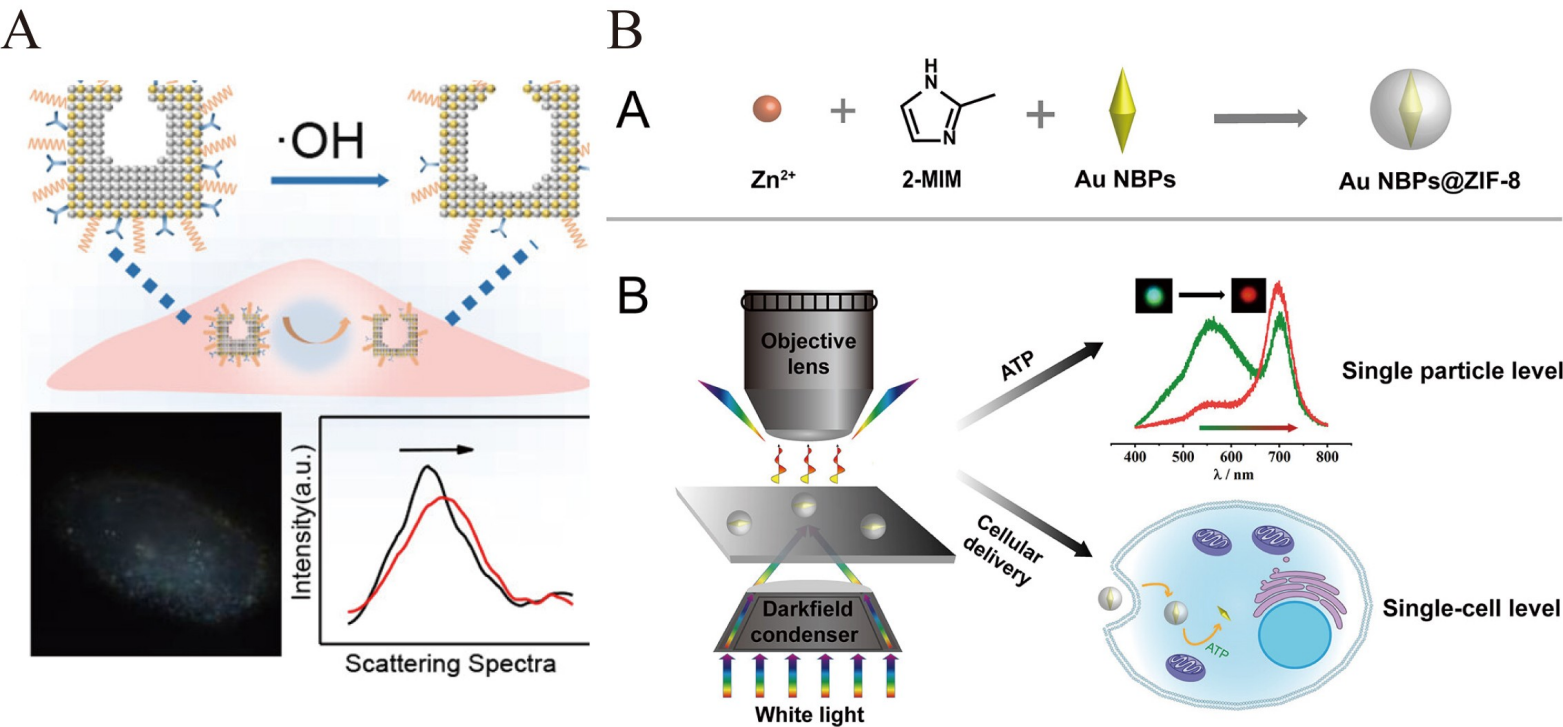
A): Construction of Ag−Au@PEG/RGD Nanocage and Diagram of the Single Particle DFM Method for Intracellular ^⋅^OH Assay. Reprinted with permission from Wu, S. et al. Anal. Chem. 92, 9940–9947 (2020).^[46]^ Copyright 2020 American Chemical Society. B): llustration of a AuNBPs@ZIF‐8 Composite Nanoprobe forExtracellular andIntracellular ATP Assay. Reprinted with permission from Chen, X. et al. Anal. Chem. 94, 18107–18113 (2022).^[47]^ Copyright 2022 American Chemical Society.

Zhu et al. prepared Ag−Au@PEG/RGD nanocages for monitoring dynamic ^⋅^OH levels in individual tumor cells under oxidative stress^[53]^(Figure 5A). Since ^⋅^OH can highly selectively oxidize the conjugated PEG/RGD molecules and the silver shells inside the nanocages, leading to significant LSPR signals and scattering color changes of the probes, it is helpful to further investigate the intracellular homeostasis and damage mechanisms mediated by ^⋅^OH.

Chen et al. outsourced AuNBPs@ZIF‐8 in a gold nanobipyramid as a probe for monitoring the fluctuation of ATP levels in living cells^[54]^ (Figure 5B). Since the coordination of ATP with Zn^2+^ is much stronger than that of 2‐methylimidazole with Zn^2+^, it leads to the decomposition of the ZIF‐8 shell layer in the presence of ATP and the exposure of the AuNBP, which causes the change of particle color in the dark field.

Xu et al. pre‐modified the AuNP surface with azide and alkyne groups, and then utilized the feature that H_2_S can selectively reduce azide to amino group to inhibit the clicking reaction, which led to a good dispersion of the AuNPs in solution; otherwise, the AuNPs underwent aggregation. The scattering color of individual AuNPs can be easily distinguished from that of aggregates of AuNPs by DFM, which enables the detection of H_2_S, an important physiological molecule.^[55]^

### 5.2 Applications Related to Microorganisms

In addition to realizing sensitive and rapid detection of proteins, nucleic acids and other biomolecules mentioned above, dark‐field microscopy has a wide range of applications in biomedical fields, which also include microorganisms, such as bacteria and viruses. Researchers have gradually realized the efficient detection of many common clinical microorganisms, such as Streptococcus, Staphylococcus aureus, Escherichia coli, as well as ultra‐small viruses such as EV71 and hepatitis viruses.

**Figure 6:**
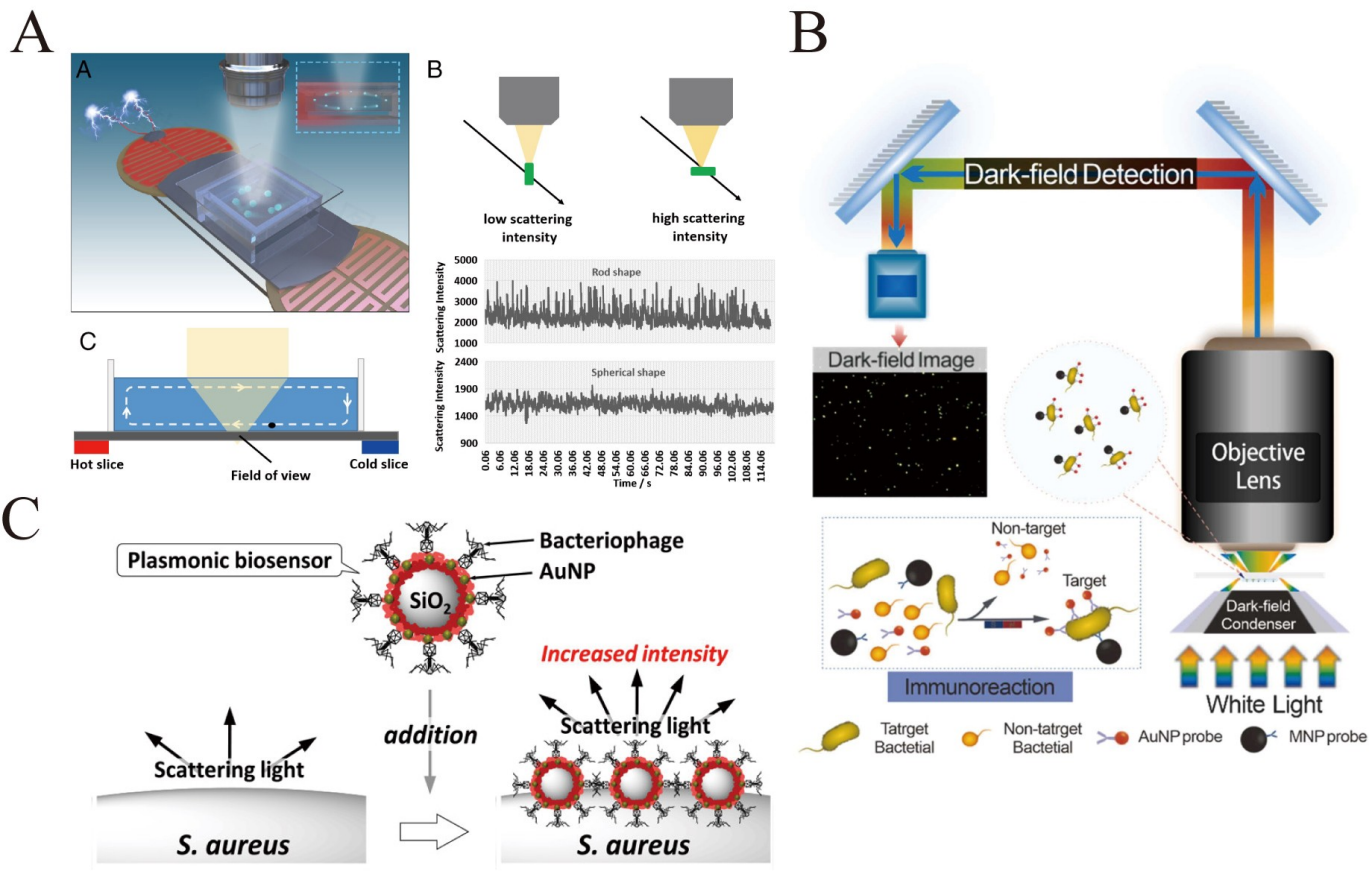
A): Bacteria detection principle by a single‐particle imaging approach. PNAS 2022 119 (40) e2206990119.^[49]^ Copyright 2022 the National Academy of Sciences. B): Schematic of the colorimetric sensor technology for the detection of E. coli O157:H7 based on dark‐field microscopy and spectral angle mapping. Reprinted with permission from Zheng, L. et al. Sensors and Actuators B: Chemical 367, 132042 (2022).^[53]^ Copyright 2022 Elsevier. C): llustration of detecting bacteria using bacteriophage immobilized SiO_2_@AuNP Core−Shell Nanoparticles. Reprinted with permission from Imai, M. et al. Anal. Chem. 91, 12352–12357 (2019).^[54]^ Copyright 2019 American Chemical Society.

Fang's group has innovatively proposed a method to differentiate positive samples of group B streptococcus (GBS) from vaginal swabs within 10 minutes^[56]^ (Figure 6A). Unlike other work on identifying streptococci, this work traces the change in scattering intensity of single particles in free solution, and through statistics and comparisons, the morphological identification of individual bacteria can be achieved in a label‐free manner at particle sizes smaller than the diffraction limit. Zhou et al. used two types of equipartitioned exciton probes: silver nanoparticles (SNPs), which appear blue in the dark field, are modified with antibodies for capturing target viruses, and gold nanorod probes (GNPs), which appear red in the dark field, form a bicolor sandwich structure with SNPs.^[57]^ Subsequently, the captured dark‐field images were processed and screened using AI algorithms to achieve rapid counting of the ultra‐small virus EV71. Wang's group developed a sensor for hepatitis B virus detection based on surface etching of spiral gold nanorods at the single‐particle level under dark‐field microscopy. Spiral gold nanorods have high surface activity, and the etching produces significant color and light scattering changes in individual particles, resulting in more sensitive detection results than ordinary gold nanorods.^[58]^ Cheng et al. developed another probe for hepatitis viruses: gold nanorods (red light‐scattering) and gold nanospheres (green light‐scattering) were attached to the surface of magnetic beads, respectively, and planetary satellite structures were constructed by hybridizing them with the corresponding capture strands (CpHBV and CpHCV), which were then specifically digested by using Nucleic Acid Ectonuclease III in order to release the two target DNA strands and the gold nanoparticles with different light‐scattering colors. particles. Simultaneous detection of HBV and HCV can be achieved by simply counting the number of red and green light scattering spots.^[59]^

Zheng developed a visualization method based on spectral angle mapping (SAM) and dark‐field microscopy for the rapid detection and quantification of Escherichia coli O157:H7^[60]^ (Figure 6B). The target bacteria were efficiently enriched by preparing immunomagnetic nanoparticles and gold nanoparticles to form a sandwich complex, and then the colonies were counted by dark‐field microscopy with a detection limit of 61 CFU/mL. The results showed that the immunomagnetic nanoparticles and gold nanoparticles were highly enriched in the target bacteria.

Imai et al. immobilized phage S13′ outside SiO_2_@AuNP core‐shell nanoparticles as a probe for specific recognition detection of Staphylococcus aureus, and SiO_2_@AuNP nanoparticles were used to enhance the light‐scattering intensity of the target bacterium, and S. aureus detection was accomplished within 15–20 min, with a limit of detection of 8×10^4^ colony‐forming units per milliliter^[61]^ (Figure 6C). By varying the phage used, this method has the potential to expand its application to specific, sensitive and rapid detection of any bacteria.

### 5.3 Applications Related to Revealing Reaction Mechanisms

**Figure 7:**
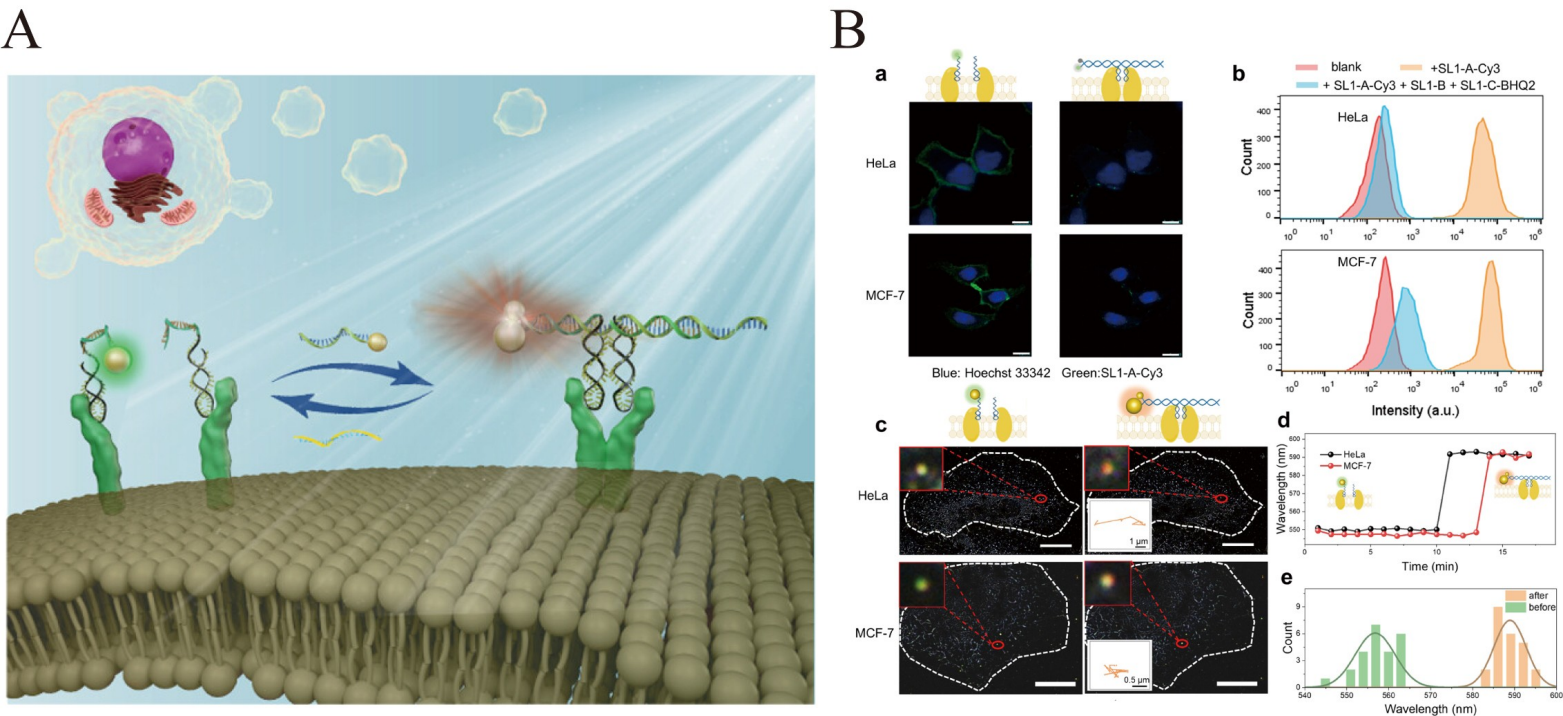
A): Principle of reprogramming and visualizing of single MET by aptamer recognition, hybridization reaction, andtoehold‐mediated DNAstrand displacement. B): MET receptors crosslink ontheliving‐cell membrane. Reprinted with permission from Wang, J. et al. J.Am.Chem. Soc. 2023, 145,1273–1284.^[55]^ Copyright 2023 American Chemical Society.

**Figure 8:**
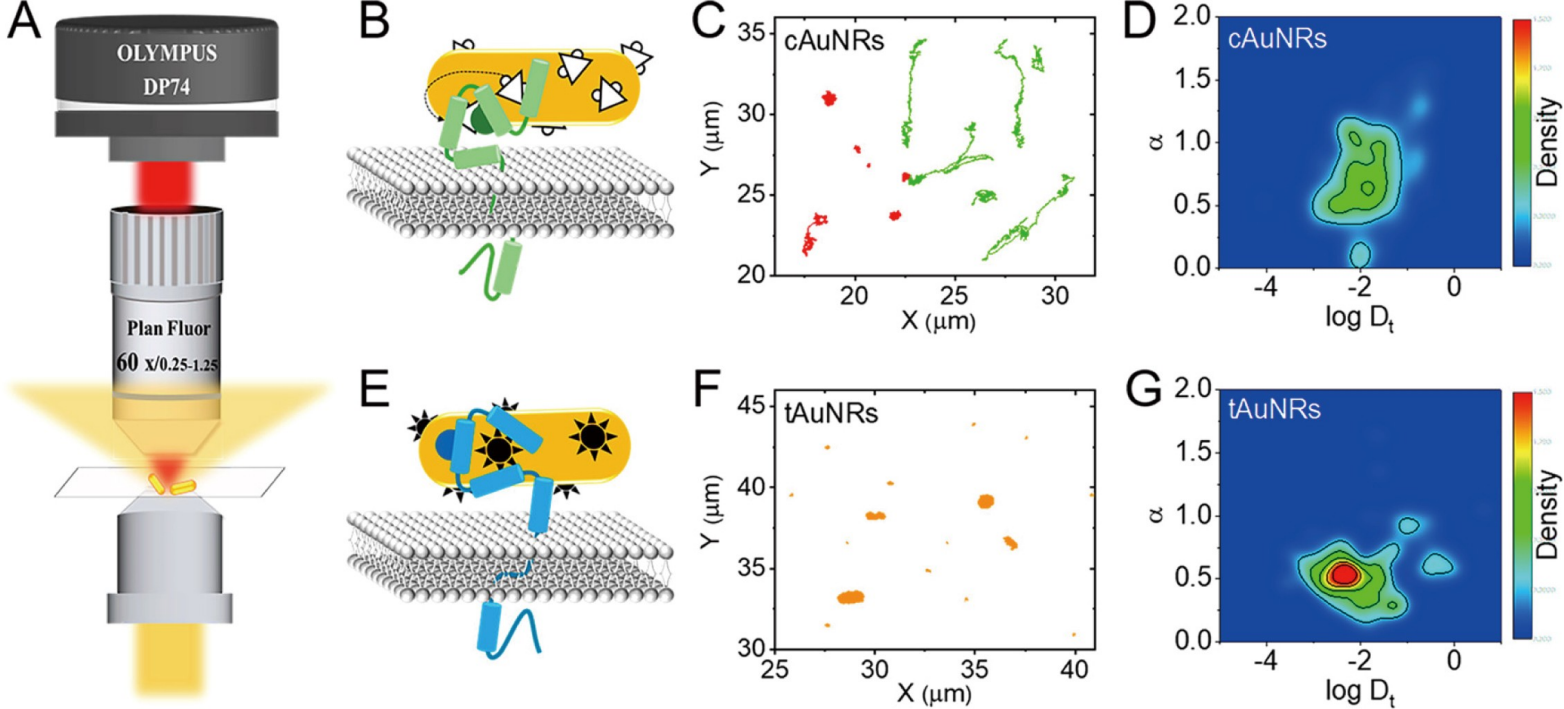
Difference in the endocytosis dynamics of anti‐EGFR modified cAuNRs and anti‐ErbB2 modified tAuNRs. Reprinted with permission from Ge, F., Du, Y. & He, Y. ACS Nano 2022, 16, 5325–5334.^[57]^ Copyright 2022 American Chemical Society.

Xu's group used DNA hybridization to induce dimerization of MET receptors on cell membranes, achieving real‐time observation of plasma‐based receptor tyrosine kinase families (RTKs) at the single‐molecule level^[62]^ (Figure 7A and B), providing new clues to reveal the kinetic study of receptor dimerization on cell membranes. Guo et al. developed a facile method to form plasma dimers with significant plasmonic resonance coupling effects and stronger scattering efficiencies in the presence of the target when azide‐ or alkyne‐modified AuNPs are introduced as probes, inducing the “turn‐on” phenomenon, which monitors the azide‐alkyne cycloaddition clicking reaction on Cu^+^‐catalyzed AuNPs on the single‐particle level.^[63]^ It is worth noting that when HER2 antibody is connected to the probe, dark‐field in situ imaging of biomolecules on cell membrane can also be achieved. He et al. investigated the dynamic endocytosis process of single gold nanorods (cAuNRs and tAuNRs) modified against Erb B by dark‐field imaging and single‐nanoparticle tracking^[64]^ (Figure 8). By tracking and analyzing the translational and rotational diffusion of single AuNRs, it was found that the different properties of the EGFR and ErbB2 receptors lead to different motility behaviors of cAuNRs and tAuNRs as well as a slower endocytosis rate of tAuNRs than cAuNRs. The above findings provide a reference for antibody‐based cancer therapy research.

## 6 Emerging Technology Combination

Dark‐field microscopy, as an optical microscope with high sensitivity, high temporal and spatial resolution, and high signal‐to‐noise ratio, is capable of single‐particle horizontal imaging. It is also non‐destructive, requires no staining or fixation, requires no labeling, and introduces no unknown interference from foreign substances. These advantages make dark‐field microscopy often used as a powerful tool in combination with other techniques to amplify each other's advantages and complement each other.

### 6.1 Surface‐Enhanced Raman Scattering

Since both plasma resonance Rayleigh scattering and surface‐enhanced Raman scattering can use plasma nanoparticles with locally enhanced electromagnetic fields as probes, it is easy to combine these two homologous techniques.

**Figure 9:**
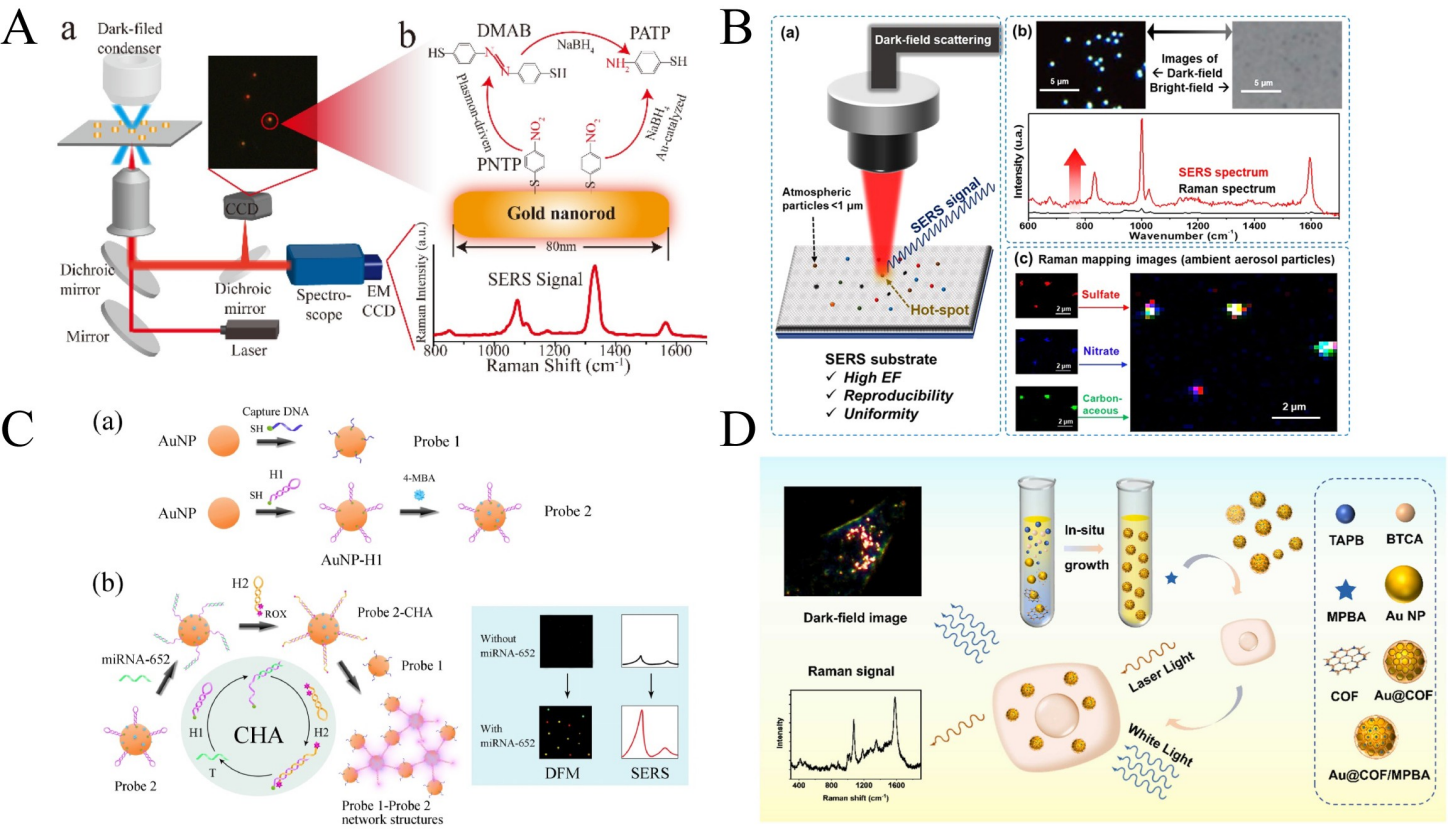
A): Schematic overview of the DFSERS. Reprinted with permission from Liu, S., Ying, Y. & Long, Y. Chinese Chemical Letters 31 (2020) 473–475.^[58]^ Copyright 2020 Elsevier. B): Schematic of detecting submicron atmospheric aerosols by combining dark‐field scattering and surface‐enhanced raman spectroscopy. Reprinted with permission from Yoo, H., Lee, H., Park, C., Shin, D. & Ro, C.‐U. Anal. Chem. 2022, 94,13028–13035.^[59]^ Copyright 2022 American Chemical Society. C): Schematic diagram of SPR/SERS dual‐mode plasmonic biosensor based on CHA‐induced AuNP network for the detection of miRNA‐652. Reprinted with permission from Song, C. et al. Biosensors and Bioelectronics 190 (2021) 113376.^[60]^ Copyright 2021 Elsevier. D): Schematic illustration of Au@COF nanoparticle for detection of intracellular metabolite through dual‐mode SERS/Dark‐field scattering imaging technique. Reprinted with permission from Tan, Z., Zhu, C., Han, L., Liao, X. & Wang, C. Sensors and Actuators: B. Chemical 373 (2022) 132770.^[61]^ Copyright 2022 Elsevier.

Liu et al. used dark‐field microscopy as an auxiliary imaging tool, which can quickly locate and adjust the position of individual AuNRs in the slit of the spectrometer, and subsequently adjust the position of individual Au nanorods into the laser spot, realizing the probing of the SERS activity of individual Au nanoparticles^[65]^ (Figure 9A). Ro et al. prepared a substrate with both surface roughness and homogeneity by sputter coating method to achieve both Raman signal enhancement and provide a clear background for dark‐field imaging, which enabled the detection of individual submicron‐sized atmospheric particles in the dark field and SERS^[66]^ (Figure 9B). The dark field enables precise visual identification of individual particles, and information on the mixing state, molecular species, and chemical functional groups of individual aerosol particles can be obtained through SERS.Wang's group reports a dual‐mode plasma biosensor using dark‐field imaging and surface‐enhanced Raman detection. After the clever design of catalytic hairpin assembly, the probe 1 and probe 2 were connected to form a gold nanoparticle network^[67]^ (Figure 9C). Probe binding significantly amplifies the SERS intensity of the two Raman reporter molecules embedded on it. At the same time, the particle size becomes larger when the particles are combined with each other, which makes their scattering intensity in the dark field also increase significantly. The respective advantages of the two modes complemented each other and assisted each other, realizing the sensitive detection of cancer‐associated microRNA‐652 with a detection limit of 42.5 fM and good and accurate recovery in human blood samples. Wang et al. reported an Au@COF core‐shell nanosensor for in situ monitoring of cellular metabolite H_2_O_2_ with the help of dual modes of SERS and dark‐field scattering^[68]^ (Figure 9D). The signaling molecule induces a change in the fingerprint level of the Raman peaks, which provides rapid insight into the intracellular concentration of hydrogen peroxide; the dark‐field imaging provides the specific intracellular distribution of hydrogen peroxide. the combination of the superior recognition capability of SERS and the imaging capability of dark‐field microscopy provides even richer optical information.

### 6.2 Fluorescence

**Figure 10:**
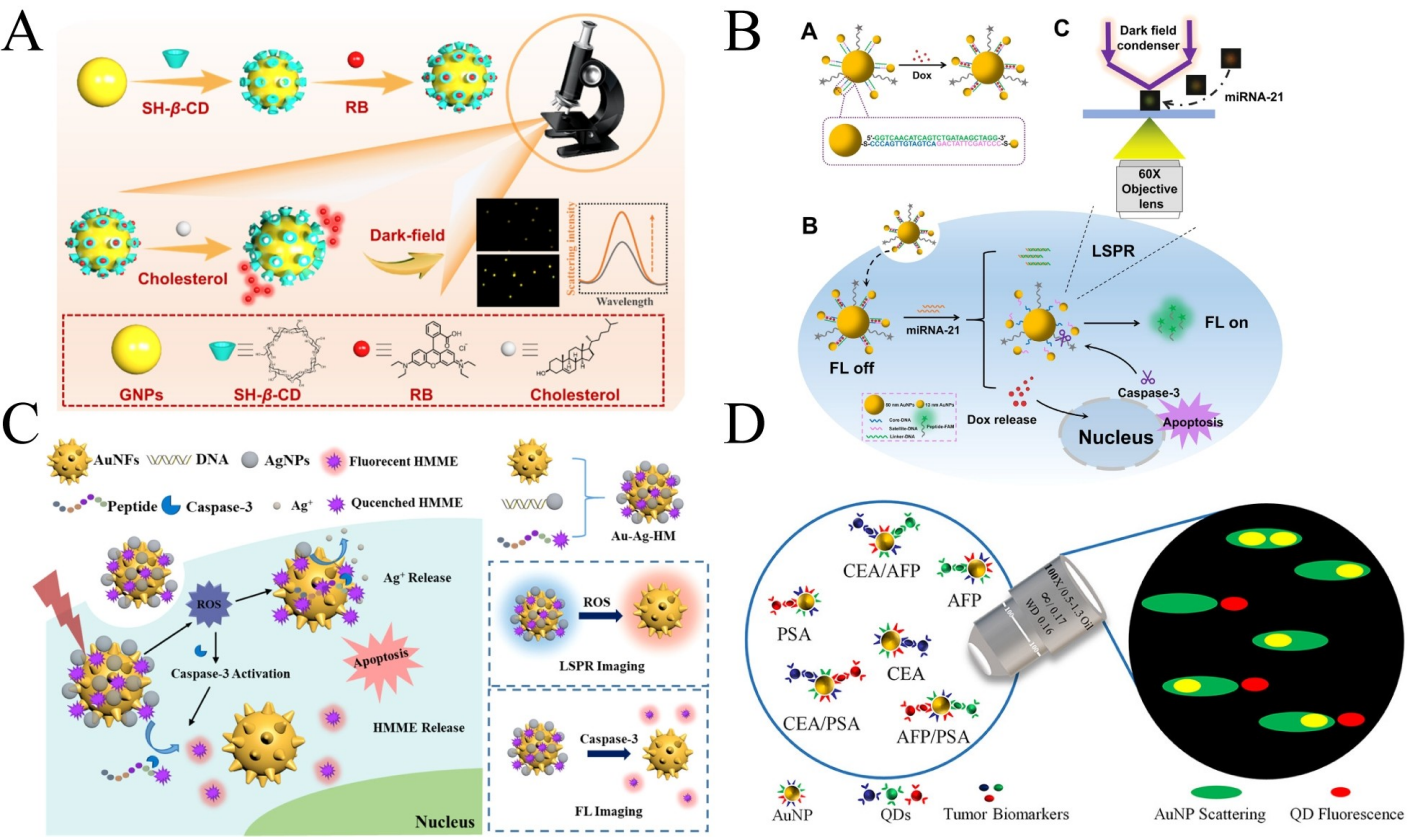
A):Schematic illustration of single‐particle detection of cholesterol. Reprinted with permission from Wang, S.‐M., Wang, H., Zhao, W., Xu, J.‐J. & Chen, H.‐Y. Copyright 2023 Elsevier.Chinese Chemical Letters 34 (2023) 108053.^[62]^ B): Scheme of intracellular miRNA‐21‐triggered disassembly of the CS nanoprobe for drug release and apoptosis imaging. Reprinted with permission from Zhang, D., Wang, K., Wei, W., Liu, Y. & Liu, S. Anal. Chem. 2021, 93, 9521–9530.^[63]^ Copyright 2021 American Chemical Society. C): Schematic Illustration of the Au−Ag−HM Nanoprobe for Cancer Cell Diagnosis and Therapy. Reprinted with permission from Wang, K. et al. Anal. Chem. 2021, 93, 7870–7878.^[64]^ Copyright 2021 American Chemical Society. D): Schematic illustration of multiplexed homogeneous immunoassay. Reprinted with permission from Liu, X., Lin, X., Pan, X. & Gai, H. Anal. Chem. 2022, 94, 5830–5837.^[65]^ Copyright 2022 American Chemical Society.

Xu's group recently reported a nanoprobe combining fluorescence and dark‐field technologies for quantitative detection of cholesterol levels^[69]^ (Figure 10A). The PRET effect occurs by modifying β‐cyclodextrin outside the gold spheres, and the lumen of the cyclodextrin carries the rhodamine molecule. In the presence of cholesterol, which binds more tightly and strongly to the cyclodextrin molecules, the rhodamine molecules are competed down and their fluorescence is restored. The PRET effect is also suppressed, and the scattering intensity of the gold spheres in the dark field is restored to increase. The results of both methods are very sharp and easy to observe. And the results of both methods can be corroborated with each other to improve the accuracy and sensitivity. Wei et al. prepared nucleo‐satellite nanoparticles with 50 nm gold spheres as nuclei and 13 nm gold as satellites for cellular microRNA imaging, drug delivery, and apoptosis monitoring. miRNA‐21 triggered the disassembly of the nucleo‐satellite structure, releasing Dox embedded in a GC‐rich double‐stranded strand. miRNA‐21 was thus detected at the single‐particle level by the LSPR technique. On the other hand, the release of the anticancer drug doxorubicin induced apoptosis and calpain I activation in cancer cells, which could be detected by fluorescence^[70]^ (Figure 10B).

Liu et al. constructed an Ag−Au‐HM nanoprobe with silver nanospheres loaded on gold nanoflowers as well as the photosensitizer HMME^[71]^ (Figure 10C). The silver nanoparticles and the photosensitizer HMME can induce apoptosis of cancer cells and oxidatively etch AgNPs, resulting in a significant change in the LSPR signal of the nanoparticles; at the same time, the apoptosis of cancer cells activates caspase‐3, which specifically cleaves the DEVD peptide on the probe, releases HMME, and restores fluores fluorescence. This probe realized both LSPR detection of ROS and fluorescence detection of caspase‐3.

AuNPs with a particle size of 70 nm were used as scattering markers and QD 525, QD 585 and QD 655 as fluorescent markers by Gai et al. A triple homogeneous digital immunoassay was established by dark‐field and fluorescence microscopy to distinguish nucleo‐satellite structural immunocomplexes from unbound and non‐specific binding probes^[72]^ (Figure 10D). The overlapping scattering and fluorescence spectral images enable precise identification and localization of immune complexes, with detection limits in the teens of femtomoles for CEA, AFP, and PSA.

### 6.3 Colorimetry

**Figure 11:**
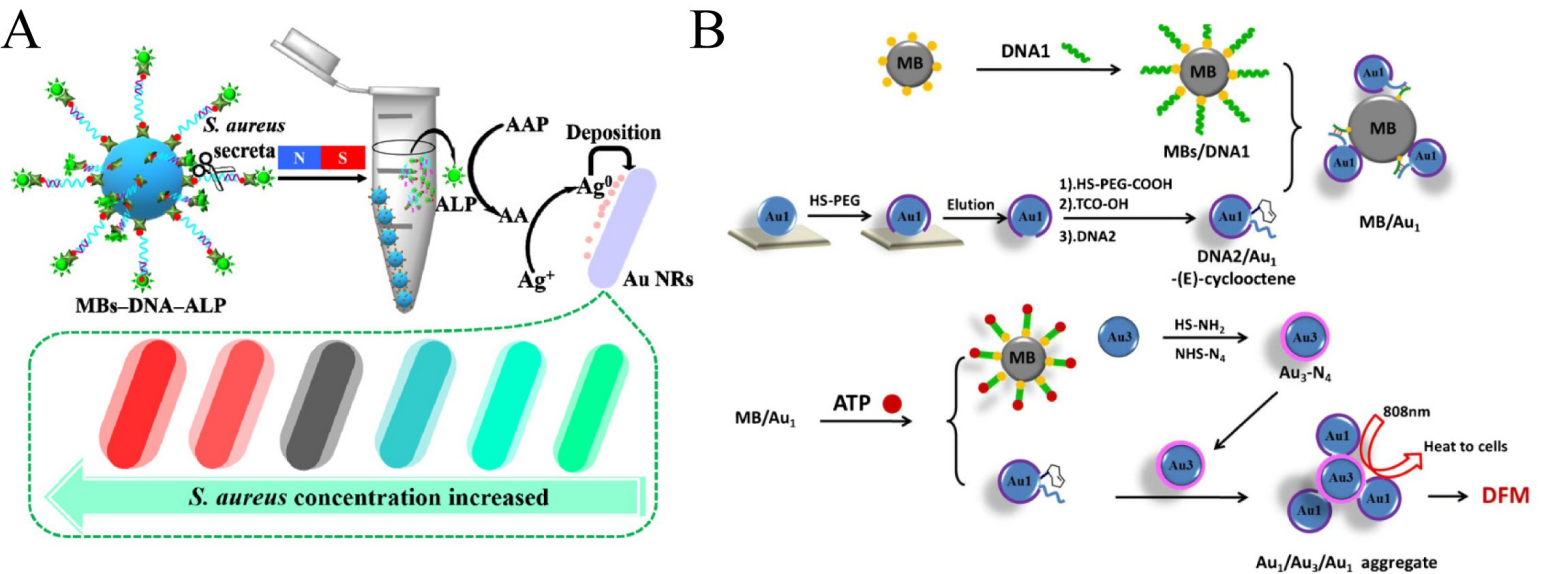
A): Mechanism of the Dual Enzyme‐Induced Au−Ag Alloy NRs as Colorful Chromogenic Substrates for the Determination of S. aureus. Reprinted with permission from Zhou, J. et al. ACS Appl. Bio Mater. 2020, 3, 6103–6109.^[66]^ Copyright 2020 American Chemical Society. B): Schematic of the tetrazine/trans‐cyclooctene cycloaddition reaction to form AuNP aggregates, which were employed in the present work for ATP detection and photothermal cancer therapy. Reprinted with permission from Liu, F., Guo, Y., Hu, Y., Zhang, X. & Zheng, X. Analytical and Bioanalytical Chemistry (2019) 411 : 5845–5854.^[67]^ Copyright 2019 Springer.

He et al.synthesized gold‐coated silver nanorods by double enzyme induction, and realized the sensitive detection of Staphylococcus aureus by combining dark field imaging and colorimetric method^[73]^ (Figure 11A). In the presence of Staphylococcus aureus and its microsphere nuclease, the specific site of DNA is sheared and alkaline phosphatase is released into solution, hydrolyzing the ascorbyl phosphate substrate to produce ascorbic acid, which in turn reduces the silver ions to form silver‐coated gold nanorods that show a difference in color, whereby the visual detection of Staphylococcus aureus is achieved.

Liu et al. developed an ATP assay based on a cycloaddition reaction, combining colorimetric methods with dark field microscopy^[74]^ (Figure 11B). They utilized two asymmetrically modified gold nanoparticles: tetrazine‐functionalized gold nanoparticles and asymmetrically modified trans‐cyclooctene‐gold nanoparticles. In this method, the presence of ATP indirectly enables the formation of AuNP aggregates with enhanced scattering efficiency from these two functionalized AuNPs via a cycloaddition reaction, enabling visual quantification of ATP and without the need for any catalyst.

### 6.4 Electrochemistry

**Figure 12:**
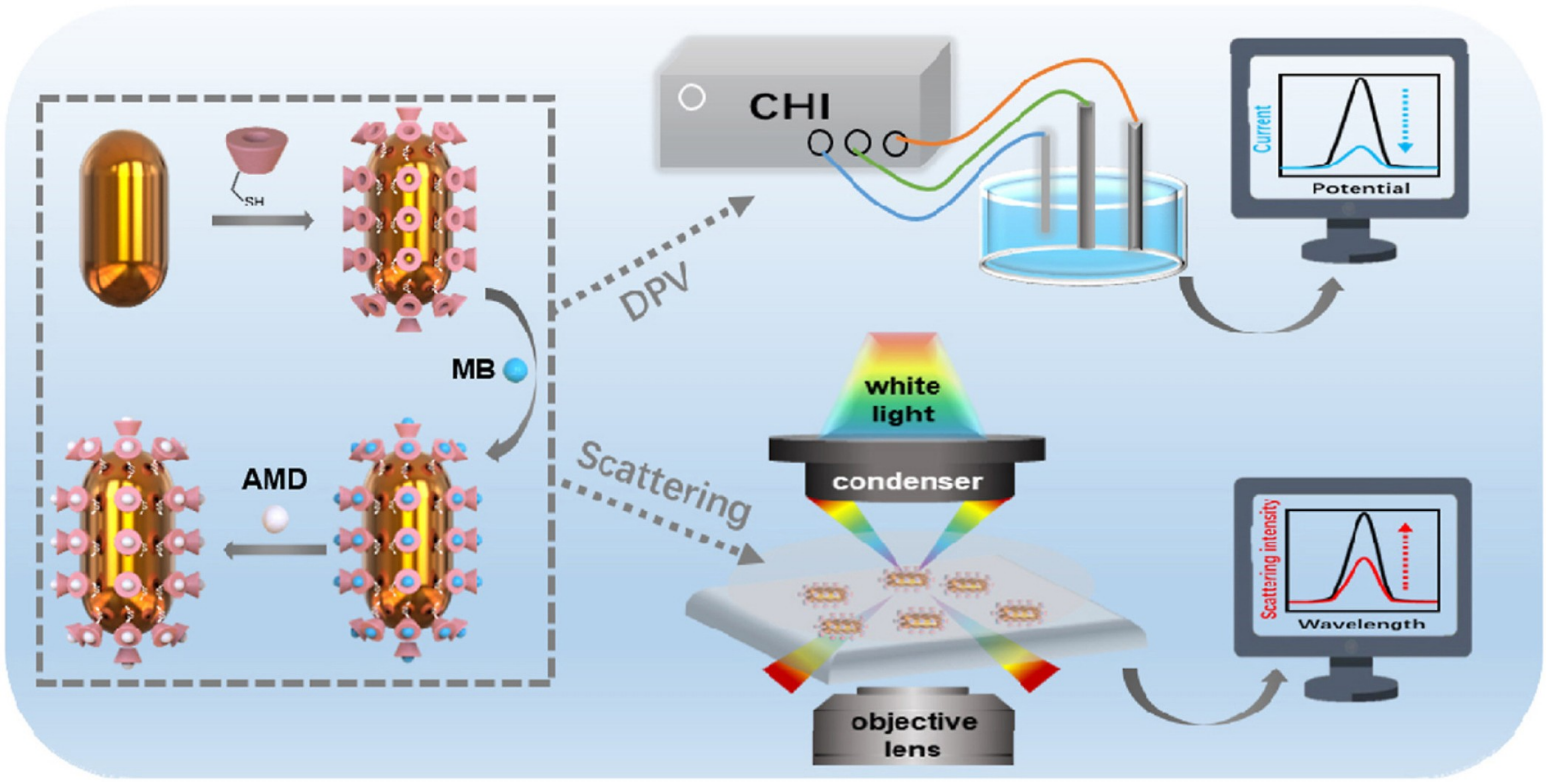
Schematic illustration of Au NR@SH‐CD‐MB NPs for dual mode AMD detection. Reprinted with permission from Wang, H., Wang, S.‐M., Zhao, W., Chen, H.‐Y. & Xu, J.‐J. Analytica Chimica Acta 1209 (2022) 339838.^[71]^ Copyright 2022 Elsevier.

In addition to the above techniques commonly used in biomedical detection, the single‐particle imaging function of the dark field and the spectral recording function also make it a great advantage when used in conjunction with electrochemistry, providing good temporal and spatial resolution for electrochemistry.^[75]^ When appropriate stimuli, such as light,laser, electric potential, or reactant,[^76^, ^77^] are applied, the evolution of the DFM image is transformed into a series of spectra as the electrochemical reaction proceeds,^[78]^ thereby quantifying changes in chemical information on individual nanoparticles.^[79]^ Xu et al.designed a probe that attached cyclodextrin to the outside of gold nanorods and inserted methylene blue into its cavity to detect the concentration of the antiviral drug amantadine^[80]^ (Figure 12). The spectral overlap between AuNR and MB molecules and the electrochemical conversion activity of MB molecules induce plasma resonance energy transfer (PRET) process and electrochemical signaling response. And when amantadine competes with methylene blue for the cavity on cyclodextrin, the scattering intensity of Au NR is restored and accompanied by the decrease of methylene blue electrooxidation current, which leads to the highly sensitive quantification of amantadine under the dual‐signal channel.

## 7 Conclusions and Perspectives

In this work, the biomedical applications of plasmonic nanoparticles in dark field microscopy scattering imaging under LSPR effect in recent years are reviewed. This work outlines the basic principles of detection, common detection mechanisms, summarizes several classic practical applications, and also lists several currently emerging technology associations, which provides a more detailed overview of the current status of dark‐field microscopy applications in the biomedical field. At the same time, perspectives on the future trends and challenges in this field are also proposed as follows.

1. The ultimate application goal of dark‐field microscopy is to solve problems in real samples. How to realize the detection of targets quickly, efficiently and conveniently is one of the future endeavors of dark‐field imaging technology. Despite the high sensitivity of the Localized Surface Plasmon Resonance (LSPR) technique for individual nanoparticles, however, it is quite time‐consuming to utilize a spectrometer to capture the spectral signals of individual nanoparticles. This process can only be acquired for one nanoparticle at a time, limiting the speed of analysis. Therefore, in the future, the development of new digital analysis techniques will be an inevitable trend to achieve rapid, high‐throughput analysis of individual nanoparticles. Several researchers are now working on the development of data processing methods combined with intelligent systems and machine learning, with the aim of realizing rapid extraction and readout of information for automated analysis. Song et al. proposed a computational strategy based on a deep learning framework (Nano Net), which will realize the effective segmentation of scattered light spots in diffraction‐limited DFM images to obtain high‐resolution iso‐excitation light scattering imaging, and successfully produced high‐resolution DFM images of living cells, which opens up a new pathway for high‐resolution optical nano‐imaging.^[81]^
2. At the same time, the nature and composition of nanoparticles utilized in dark‐field scattering imaging is also an important element driving its development. In the future, dark‐field scattering imaging needs to be more closely integrated with materials science to explore materials with better performance, more economical and easier to produce, which can be used for dark‐field imaging. Single‐particle dark‐field scattering imaging has been mainly focused on precious metal nanoparticles, especially gold and silver, which limits the application areas of dark‐field microscopy to some extent. Therefore, there is a need to expand the study of the optical properties and applications of other non‐precious metal nanomaterials. The plasmonic properties of non‐precious metal nanomaterials present both challenges and opportunities for their application as nano‐probes.^[82]^ Although non‐precious metals (such as copper and aluminum) exhibit relatively weak plasmonic characteristics in the visible light region due to fewer free electrons, optimizing synthesis conditions (such as particle size and shape) can enhance their optical response, making them effective nano‐probes.^[83]^ For applications requiring deeper penetration of optical signals, emitting in the infrared region is an effective strategy. Infrared light offers better biocompatibility and penetration ability.^[84]^ Therefore, it is worth considering the synthesis of nanomaterials with infrared absorption and fluorescence emission properties, such as non‐precious metal nanoparticles doped with rare earth elements or composite materials. Despite the challenges, advancements in materials science and nanotechnology hold promise for optimizing the structure and synthesis strategies of these materials, thereby enhancing their performance in applications.
3. Although localized surface plasmon resonance (LSPR) technology has been widely used for biomarker detection and imaging, it has not yet become a clinically established early diagnostic technique due to its limited depth of tissue penetration. In order to detect and image lesion areas in tissues and organisms more deeply, methods combined with other optical techniques that require laser beams can be used to overcome the limitation of the insufficient depth of penetration of optical signals in in vivo imaging.^[85]^

## Conflict of Interests

The authors declare no conflict of interest.

8

## Biographical Information

*Yue Cao graduated from Qiqihar University in 2007(BSc) and 2011(MSc). She finished her Ph.D. studying at ECUST under the supervision of Prof. Yi‐Tao Long in 2016, following by postdoctoral research on SERS and electrochemical sensing at ECUST. In 2017, she was awarded the Hong Kong Scholars Program and worked as a postdoctoral fellow at the Chinese University of Hong Kong. She was appointed as an associate professor at NJMU in 2019. She is currently focusing on nano spectroelectrochemistry to reveal the interactions of biomolecules in single cells and the detection of forensic toxicants by SERS and electrochemistry*.

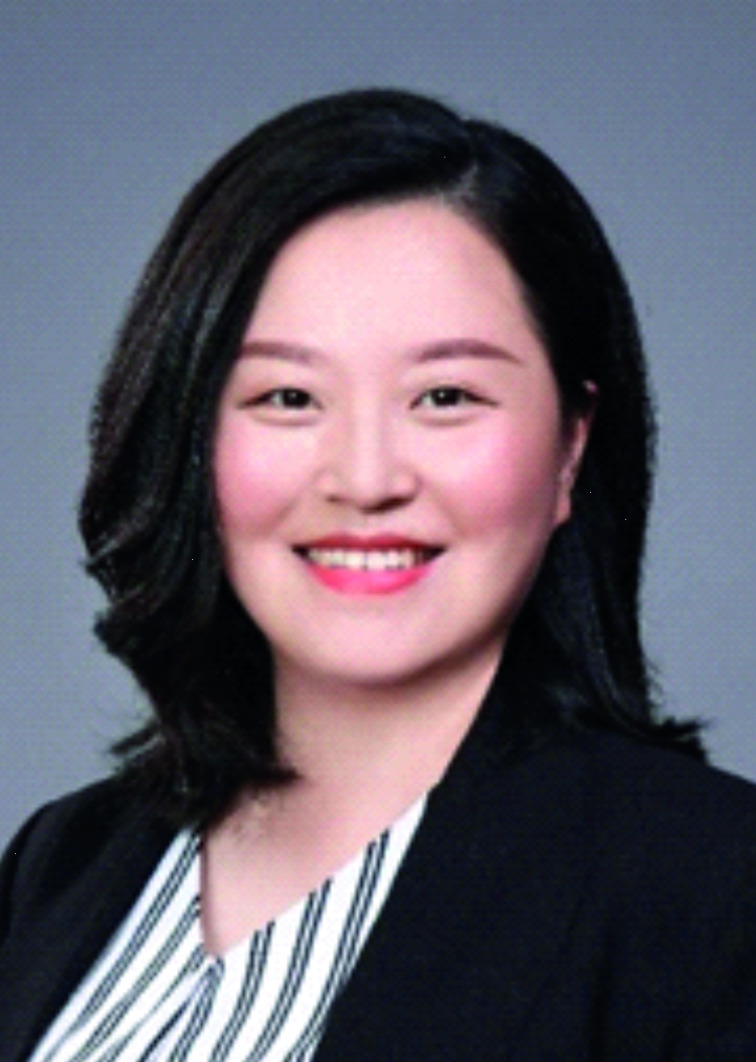

## Biographical Information

*Feng Chen received his B.M.S. from Xi′an Jiaotong University in 2005 and his Ph.D. in Forensic Medicine from Xi′an Jiaotong University in 2011. Afterward, he undertook two years of postdoctoral studies at Medical College of Georgia, Augusta, Georgia. He was appointed as a professor and director at the Department of Forensic Medicine at Nanjing Medical University in 2013. His main research expertise involves cardiovascular disease and sudden cardiac death, molecular genetics, and molecular biology*.

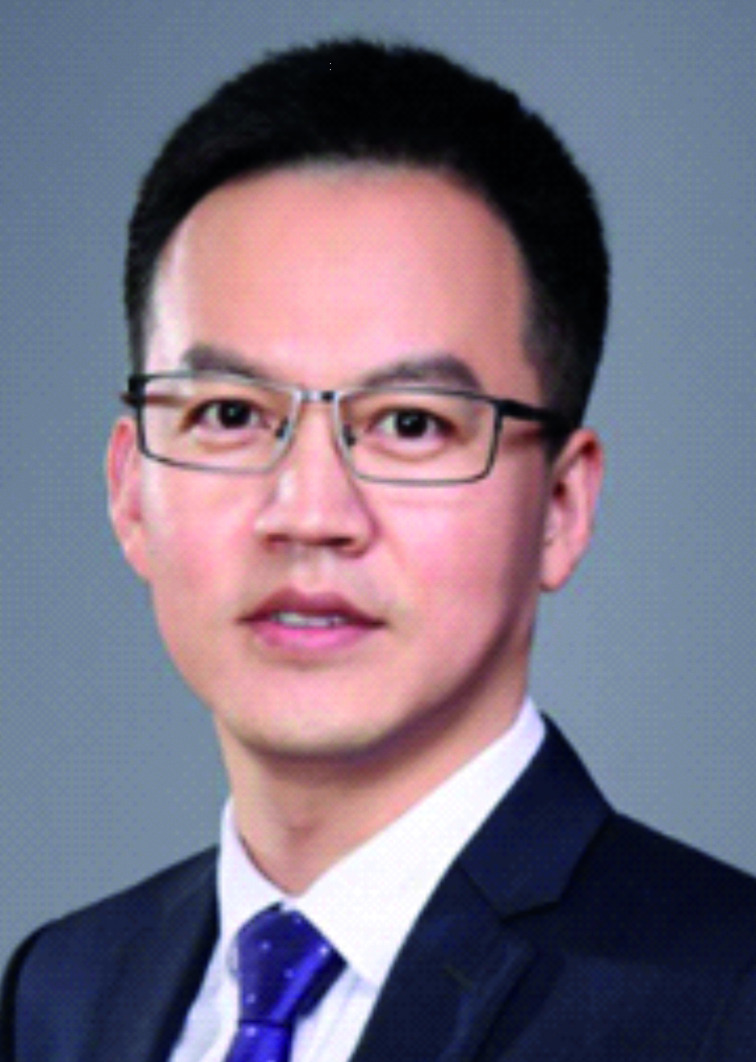

## Biographical Information

*Lingling Li is currently a professor at the School of Pharmacy, Nanjing Medical University. She received her Ph.D. degree in 2011, majoring in Analytical Chemistry, from Nanjing University. In March 2019, she was employed as a professor at Nanjing Medical University. Her main research interests are biomedical applications of optical/electrical nanomaterials, using novel optical/electrical functional nanomaterials as a tool to carry out research on relevant scientific problems in biomedical fields, including preparation of functional nanomaterials; optical/electrical nanobiosensing; and smart nano‐drug delivery systems*.

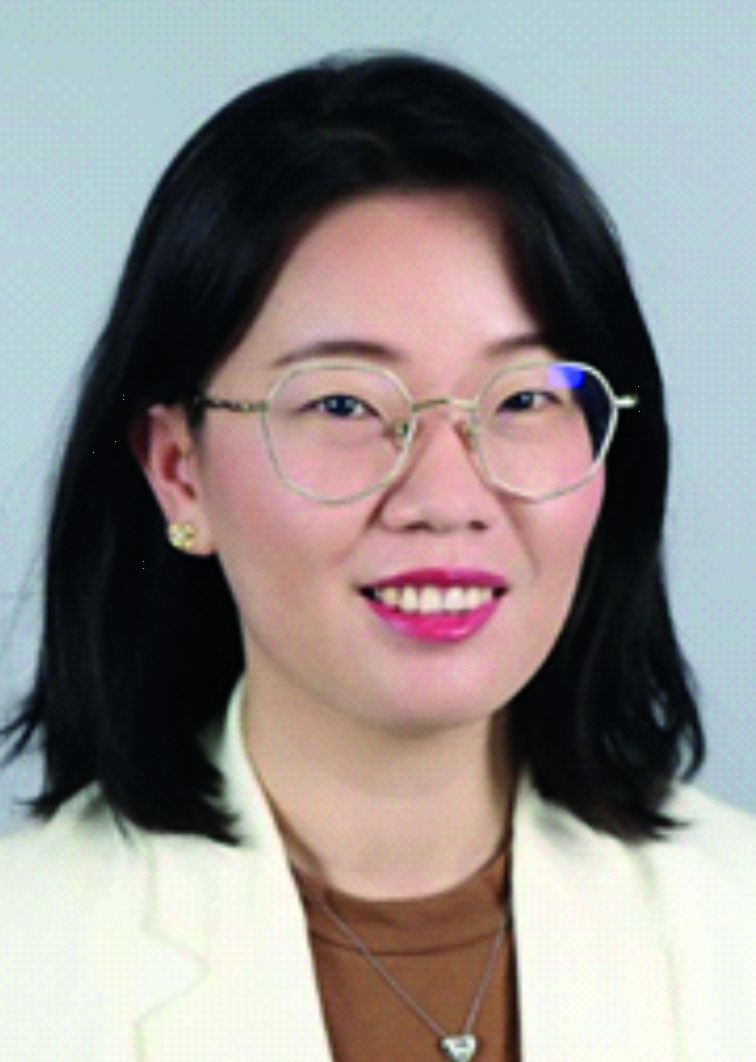

## Biographical Information

*Yang Shi is pursuing her Master of Pharmacy degree at the School of Pharmacy, Nanjing Medical University. She graduated from the Department of Clinical Pharmacy of Nanjing Medical University as an undergraduate in 2022. Now her research interests are focused on optical sensing related to nanomaterials, including novel nanomaterials synthesis, surface‐enhanced Raman scattering, and dark‐field scattering spectroscopic imaging*.

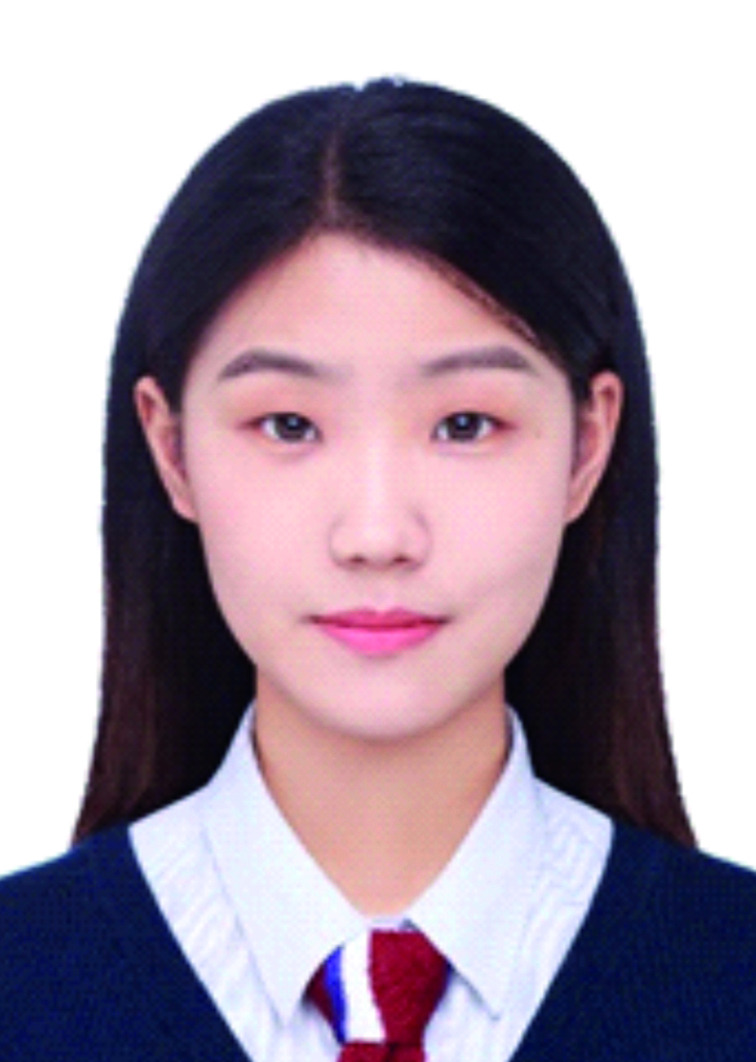

## Biographical Information

*Lixiang Wang is pursuing her Master of Forensic Medicine at the School of Basic Medical Sciences, Nanjing Medical University. She graduated from the Department of Forensic Medicine, Nanjing Medical University as an undergraduate in 2022. Her postgraduate research direction is to utilize COF‐based SERS sensors to identify multiplex VOC*.

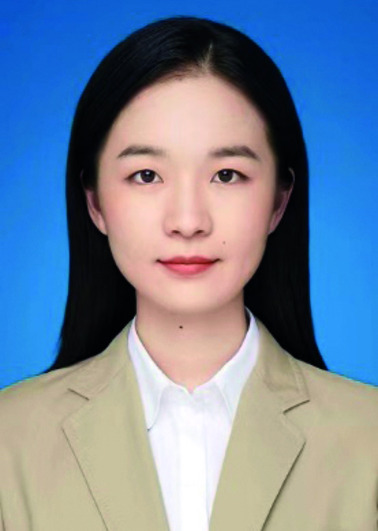

## Data Availability

The data that support the findings of this study are available in the supplementary material of this article.
